# Mind the gap: the temporal discrimination threshold of tactile sensation from implanted peripheral nerve stimulation

**DOI:** 10.1186/s12984-026-01986-9

**Published:** 2026-04-18

**Authors:** John D. Wright, Hamid Charkhkar, Emily L. Graczyk

**Affiliations:** 1 Department of Biomedical Engineering, Case Western Reserve University, Cleveland, OH 44106 USA; 2 Louis Stokes Cleveland Department of Veterans Affairs Medical Center, Cleveland, OH 44106 USA

**Keywords:** Peripheral Nerve Stimulation, Timing, Neuro-prosthesis, Sensory Restoration

## Abstract

**Background:**

The ability to reliably detect the temporal order, overlap, and gaps between multiple tactile stimuli is crucial for making sense of our haptic interactions with complex environments. One important property of temporal processing of touch is the temporal discrimination threshold (TDT), which measures the shortest gap between two sequential stimuli required for them to be perceived as separate events. While the temporal properties of natural somatosensation are well understood, much less is known about the temporal properties of sensation elicited by peripheral nerve stimulation (PNS) delivered via implanted neural interfaces. Improving our understanding of the TDT of PNS-evoked sensations is crucial for the development of effective neuroprostheses.

**Methods:**

This study was conducted with two individuals with unilateral upper limb loss implanted with 16-contact extraneural electrodes around the median, ulnar, and/or radial nerves. To measure TDT, we used an ascending staircase paradigm commonly used for clinical assessment of TDT. We investigated the effects of stimulation intensity, pulse train duration, and frequency on the TDT for five electrode contacts in total across the participants.

**Results:**

We found that the TDT significantly decreased with increasing stimulation intensity and frequency. We also found that the TDT of PNS pulse trains was significantly lower than the TDT of individual pulses. However, the duration of the stimulation pulse train did not have a consistent effect on TDT. For both participants, TDT values for implanted PNS were comparable to the values previously seen in the literature for electrocutaneous stimulation.

**Conclusion:**

Since PNS intensity and frequency are routinely adjusted to shape sensory experiences in neuroprosthetic applications, our findings highlight the need for coordinated control of stimulation timing alongside modulation of other PNS parameters. This coordination is essential to enable accurate timing perception, which is critical for functional upper limb tasks, such as detecting object slippage, manipulating objects, and maintaining grasp force. Therefore, our results have important implications for optimizing the delivery of sensory feedback in neuroprosthetic systems.

*Trial registration*: This study was prospectively registered on ClinicalTrials.gov (#NCT04947462) on 06/23/2021.

**Supplementary Information:**

The online version contains supplementary material available at 10.1186/s12984-026-01986-9.

## Introduction

Accurately perceiving and judging the timing of events is essential for many aspects of human behavior, including interpreting multimodal sensory information and performing complex motor tasks. In somatosensory processing, timing information helps us perceive textures [1, 2], identify object properties or surface characteristics by touch, perform stereognosis [3, 4], and interpret sensations of moving touch [5–8]. Human time perception spans at least 12 orders of magnitude, from milliseconds for precise perceptual tasks (smallest order of magnitude) to days or weeks for circadian timing (largest order of magnitude) [9]. The milliseconds to seconds range is especially crucial for sensorimotor control, where rapid feedback enables precise motor adjustments, as well as first order sensory perception [10].

Following limb amputation, individuals lose access to all touch information from the missing limb, which can impair their ability to perform activities of daily living and reduce their quality of life [11, 12]. Limb amputation impacts upwards of 550 million individuals worldwide, with an estimated 13 million new traumatic amputations annually [13]. Recent advances in peripheral nerve stimulation (PNS) have made it possible to restore touch sensation after limb loss, helping the user to compensate for the sensory deficits caused by the amputation [14, 15]. One effective neural interface for delivering PNS is extra-neural cuff electrodes, which are surgically implanted around the residual peripheral nerves. These electrodes can reliably produce sensations with controllable intensity [16], location [17], and quality [18], and have been shown to remain stable for over 12 years [17, 19]. These advances have enabled the development of sensory neuroprostheses, which restore somatosensory feedback by electrically stimulating peripheral nerves to convey information about the prosthesis’ interactions with the environment [15, 20].

Sensory neuroprostheses aim to provide real-time feedback about limb movements and the dynamics of object interactions in the prosthesis’ grasp. To make neuroprostheses more intuitive to use and better able to provide useful, accurate, and timely sensory feedback, it is essential to better understand the temporal properties of sensations produced by PNS. Recent studies have shown that the timing of PNS can affect visual-tactile integration [1] and texture discrimination [2]. However, one important unanswered question is how well users can interpret temporal gaps in sequential touch stimuli delivered via PNS. Detecting temporal gaps is important because it allows users to detect sequential stimuli as distinct touch events. This capability is required to perceive the order in which touches are applied to a neuroprosthesis, which is necessary for the user to interpret complex interactions with the prosthesis, such as the dynamics of multi-finger grasps, object exploration for stereognosis, and movement across a prosthesis finger, such as during texture scanning [21] or object slippage [22, 23]. Given the central role of temporal gap detection in the interpretation of sensory stimuli [18, 24] better understanding this phenomenon for PNS-elicited sensation is key to advancing intuitive and reliable feedback in neuroprostheses.

In this study, we evaluated the temporal discrimination threshold (TDT) of PNS-elicited sensation. The TDT is the minimum temporal delay, or gap, between two sequential stimuli that is reliably detected [25]. The TDT is a well-established psychophysical measure used to understand sensory processing. TDT values typically range between 20-80ms for normal touch, which is typically assessed with electrocutaneous stimulation of the finger [25]. The metric has been shown to have high repeatability and is resistant to training and memory effects [25, 26], and has high day-to-day reliability [27]. While the TDT has been extensively studied for normal touch in both healthy and clinical populations [27–31], it has not yet been assessed for sensory percepts evoked by direct stimulation of the nerves through extra-neural cuff electrodes in people with limb loss. Assessing the TDT for PNS allows us to better understand how PNS is perceived and processed on the order of milliseconds [25]. In addition, comparing the TDT for PNS in people with limb loss to the large body of literature on TDT for normal touch in healthy subjects provides an opportunity to determine how closely artificial touch replicates the temporal processing properties of intact sensation [25, 27, 30].

In this study, we investigated the TDT of touch sensation evoked by PNS delivered through 16-channel composite flat interface nerve electrodes (C-FINEs) surgically implanted around the median, radial, and/or ulnar nerves of two individuals with unilateral upper limb loss. We first measured TDTs for pairs of individual PNS pulses, which followed the established clinical method [25] and thus allowed direct comparison to the natural touch literature. In addition, because sensory neuroprostheses typically provide pulse trains that span seconds to minutes to convey sensory feedback when used for a functional task, we also measured TDT for pairs of pulse trains. We investigated the effects of stimulation intensity, pulse train duration, and pulse frequency on the TDT. These parameters were selected because they are commonly modulated to convey functionally-relevant information about object interactions during sensory neuroprosthesis use, such as the pressure exerted by the prosthesis or the grasp duration [15, 32, 33]. This research aimed to understand how well users of sensory neuroprostheses can detect the timing of PNS-elicited touch sensations, and how this ability changes under different stimulation conditions. The findings will help improve the design of neuroprosthetic systems to better align PNS paradigms with users’ temporal perceptual capabilities to promote enhanced functional use.

## Methods

**Fig. 1:**
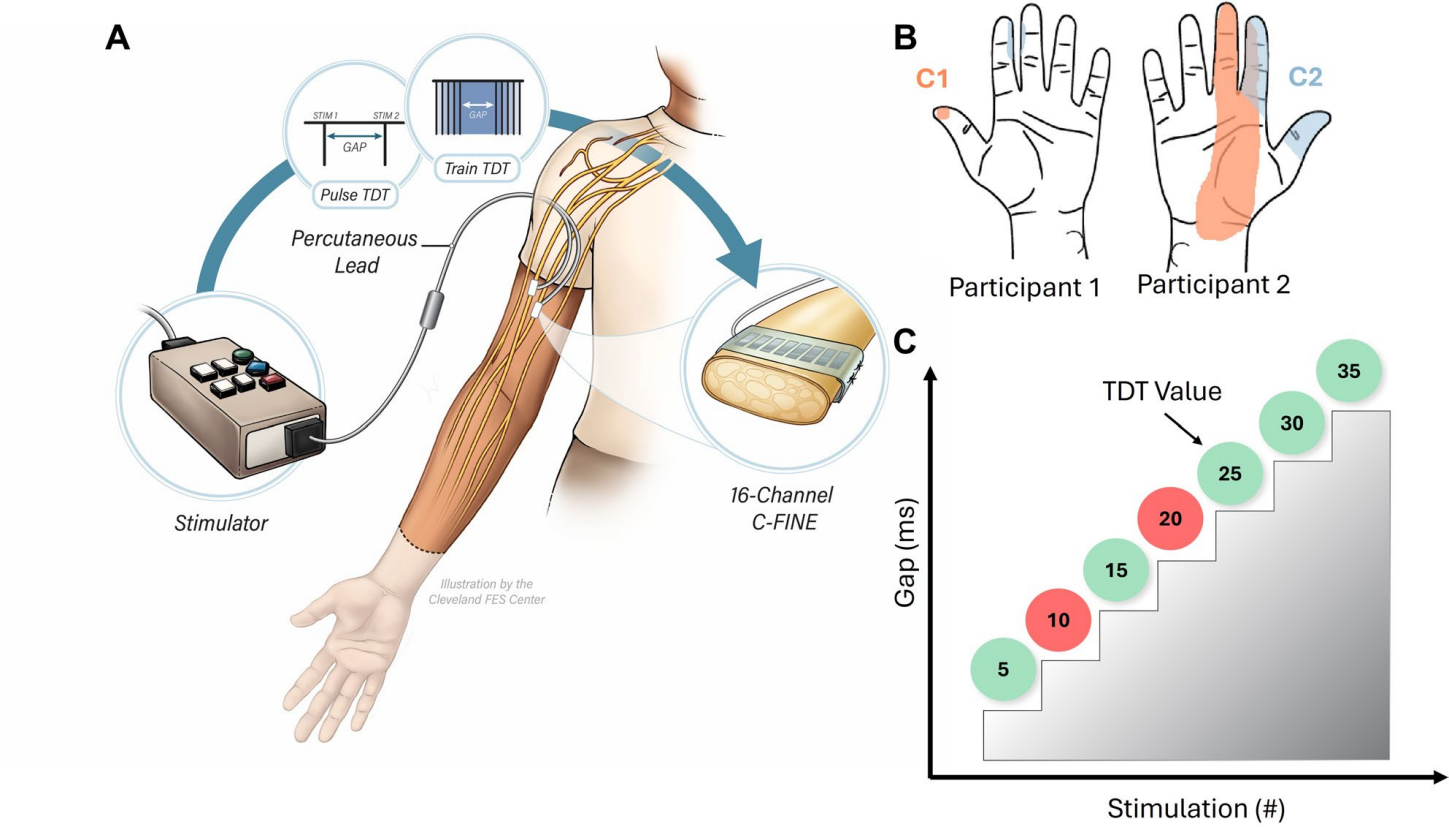
Peripheral nerve stimulation system and experimental overview. **A** Stimulation was applied from a clinical neurostimulator to individual contacts in the 16-channel composite flat interface nerve electrodes (C-FINEs) implanted around the participant’s residual nerves. Stimuli applied in the TDT experiments were either sequential pairs of individual pulses (Pulse TDT) or sequential pairs of pulse trains (Pulse TDT) separated by a variable gap. **B** Representative percept drawings for the PNS-elicited sensations in participant 1 (left) and participant 2 (right). Each participant completed the experimental tasks for two C-FINE contacts that elicited sensation on the ventral surface of the missing hand. The color indicates the C-FINE contact stimulated (C1 or C2). **C** An ascending staircase method was used to determine the TDT for PNS-elicited sensation. The gap between the stimuli in a pair incrementally increased in successive trials by steps of 5 or 10 ms until the participant correctly identified gaps in three successive trials. Correct responses are shown in green and incorrect responses are shown in red. The lowest gap value in this set of three sequential correct responses was taken as the TDT (arrow)

## Participants

Two male participants with unilateral upper limb amputation were enrolled in this study. Both had previously undergone outpatient surgical procedures in which 16-contact C-FINEs [34, 35] were installed around the median, radial, and/or ulnar nerves of the residual limb. At the time of their enrollment in the study, the participants were 5- and 10-years post-amputation and 41 and 51 years of age, respectively. Participant 1 (P1), with a left trans-radial amputation, received two 16-channel C-FINEs around the median and ulnar nerves in the upper arm. Participant 2 (P2), with a right trans-humeral amputation, had two C-FINEs placed at the most proximal segments of the median and radial nerves in the axilla. The experiments outlined in this study occurred between months 82 and 94 after C-FINE placement for Participant 1, and between months 4 and 16 for Participant 2. Participants visited the laboratory for one to three days of testing every 4–6 weeks, where each testing session lasted between 2 and 4 h, depending on their availability. All devices and procedures utilized in this study were reviewed and approved by the U.S. Food and Drug Administration under an Investigational Device Exemption (IDE #G110043) and the study protocol was approved by the Louis Stokes Cleveland Department of Veteran Affairs Medical Center’s Institutional Review Board. Written informed consent was obtained from both participants prior to enrollment.

## Peripheral nerve stimulation

Peripheral nerve stimulation (PNS) was delivered as trains of rectangular, biphasic, charge-balanced pulses with the cathodal phase leading, generated by a custom clinical-grade neurostimulator (Universal External Control Unit, Technical Development Laboratory, Cleveland, OH). Stimulation delivery was achieved using custom software designed in Simulink (Mathworks Inc., Natick, MA), executed on a high-performance real-time system (Speedgoat, Switzerland). A custom graphical user interface (GUI) designed in MATLAB (Mathworks Inc.) enabled control of the stimulation parameters during experiments. PNS was delivered through individual contacts in the C-FINEs around the participants’ median nerves, with a designated large contact within the same C-FINE serving as the return electrode (Fig. 1A). Each experiment was performed with C-FINE contacts on the median nerve (labeled C1 and C2 here). No contacts on the radial or ulnar nerves were used in this experiment. Contacts were selected that evoked tactile sensation on the missing hand and were perceptually distinguishable as two areas (Fig. 1B). For TDT experiments, stimulation was delivered as either: (1) pairs of individual pulses or (2) pairs of pulse trains, where train length varied from two to several hundred pulses, depending on the specific experiment. For the TDT experiments (see below), the time interval between the two sequential stimuli was systematically varied to assess the experimental hypotheses.

## Thresholding and dynamic range procedure

At the beginning of each testing session, the sensory threshold and dynamic range were determined for each target contact with a method of ascending limits [36, 37]. Briefly, pulse frequency (PF) was set and held at 100 Hz. A pulse amplitude (PA) between 0.5 mA and 1.5 mA was selected to evoke a detectable and comfortable sensation. The selected PA was held constant for that contact throughout the experiment. To determine sensory threshold, pulse width (PW) was initially set to 0 µs and incremented in steps of 5 µs until the participant reported perceiving an evoked sensation. The maximum comfortable limit for that contact was then determined by increasing the PW until the participant reported that the stimulation should not be increased, or until motor activation was reported by the participant or observed by the experimenter, whichever came first. The range of PW values between the sensory threshold and the maximum comfortable limit was defined as the sensory dynamic range for that contact. If a sensory dynamic range could not be established at the initial PA, the PA was increased by a step of 0.1 mA (the resolution of our stimulator), and the process was repeated. For trials with individual pulses, a maximum comfortable level was not always achievable within the range of safely deliverable charge [38]. In these cases, the PA was adjusted to ensure the PW for the sensory threshold was below 70 µs and the maximum PW was 250 us, allowing for a sufficiently large dynamic range in the subsequent experiment. This procedure was repeated for each selected contact before beginning the experimental tasks described below.

## Temporal discrimination threshold (TDT) procedure

We measured the TDT, or the point at which a gap in two sequential stimuli delivered to the same C-FINE contact can be reliably detected, using a stepwise ascending staircase paradigm, following procedures commonly used in clinical research [25, 39–43]. Briefly, each staircase began with a 0 ms gap between two stimuli. The inter-stimulus interval was then increased in subsequent trials in 5 or 10ms steps, depending on the experiment (Fig. 1C). For each trial, participants reported whether they perceived a gap between the two stimuli. Participants were instructed to report if no sensation was perceived. The staircase process continued by incrementally increasing the gaps in subsequent trials until the participant reported detecting a gap between stimuli in three consecutive trials. The first of these three gap durations was recorded as the TDT for the staircase. To ensure reliable reporting and reduce response bias, 0 ms catch trials (no gap) were randomly interspersed within the staircase procedure. Additionally, staircases would occasionally be extended to include up to five consecutive positive detections to prevent participants from identifying the criteria used to determine the TDT. In each TDT experiment, a single stimulation parameter, such as PF, PW, or train duration was varied to determine its effect on the TDT (Table 1).

**Table 1:** Summary of experiments. For each experiment (rows), the table lists the experiment number, name, stimulation paradigm, independent variable, and outcome measure

Experiment #	Experiment title	Stimulation type	Variable	Outcome measure
1.1	Pulse TDT	Pairs of Pulses	Intensity	TDT
1.2	Train TDT	Pairs of Pulse Trains	Intensity	TDT
1.3	Detectability	Single Pulses, Single Pulse Trains	Intensity	Detection
2.1	TDT of Pulses and Short Pulse Trains	Pairs of Pulses, Pairs of Pulse Trains	Train Duration	TDT
2.2	Train Duration and TDT	Pairs of Pulse Trains	Train Duration	TDT
3.1	Frequency and TDT	Pairs of Pulse Trains	Pulse Frequency	TDT
3.2	Frequency Estimation Task	Single Pulse Trains	Pulse Frequency	Continuity Rating

## Exp 1. Effect of stimulation intensity on TDT

### Exp 1.1 Pulse TDT

We first examined the effect of stimulation intensity on the TDT between pairs of single pulses delivered via a single C-FINE contact. For each contact, the sensory dynamic range was found utilizing the ‘Thresholding and Dynamic Range’ procedure described above. Three stimulation intensity levels corresponding to approximately 10%, 50%, and 90% of the sensory dynamic range were selected, where the PA was held constant, and PW was varied to achieve the desired intensity levels. The TDT was determined using the ascending staircase paradigm with 5 ms steps, and each gap value was presented once per staircase before advancing to the next. The three intensity levels were randomized across staircases but held constant for both pulses in the trial and for all trials in the staircase. For each session, two contacts were tested, and the staircases were randomized across contacts and intensity conditions to reduce the effect of adaptation [44]. Each intensity level was presented in 5 or 10 staircases per session, totaling 15 staircases per intensity level for each contact and participant.

### Exp 1.2 Train TDT

We then examined the effect of stimulation intensity on the TDT between pairs of 500 ms pulse trains delivered at 100 Hz (50 pulses per train). The same three intensity levels (10%, 50%, and 90% of the sensory dynamic range) were used for each contact. The TDT was determined using the ascending staircase procedure with 10ms steps, and each gap duration was repeated twice before advancing to the next step. As in the pulse TDT, intensity levels were randomized across staircases but held constant within each trial and for all trials in the staircase, and two contacts were tested per session. For each contact, we collected a total of 15 repetitions of each intensity condition.

### Exp 1.3 Detectability

We examined the participants’ ability to detect stimulation at each of the three intensity levels used in experiments 1.1 and 1.2 (i.e., 10%, 50%, and 90% of the sensory dynamic range) to investigate whether the detectability of different intensity stimuli may have influenced the participant’s TDT values. For each contact, we delivered either single pulses or 100 ms pulse trains at 100 Hz (10 pulses per train). In each trial, participants indicated whether they perceived sensation. Catch trials with no stimulation were randomly interspersed in 7% of trials. Each condition (3 intensity levels + catch) x 2 stimulus types (i.e., pulse and train)) were presented 30 times per contact, and the order of conditions was randomized.

## Exp 2. Effect of stimulus duration on TDT

### Exp 2.1 TDT of pulses and short pulse trains

To determine how stimulation duration affected the TDT, we compared four pulse train conditions consisting of pairs of 1, 2, 5 or 10 pulses delivered at 100 Hz to a single C-FINE contact per participant. These stimuli corresponded to 10, 20, 50, and 100 ms of stimulation duration, respectively. Stimulation intensity was set at approximately 90% of the sensory dynamic range for each contact. The 90% setting was selected to ensure that all pulses and brief pulse trains would be readily detectable. TDT was determined using an ascending staircase with 5ms steps and a single repetition before proceeding. Each condition was tested a total of 15 times per contact, and conditions were presented in randomized order.

### Exp 2.2 Train duration and TDT

We next evaluated how the duration of pulse trains affected the TDT. Stimuli consisted of 100 Hz pulse trains lasting 100, 500, and 1000 ms, corresponding to 10, 50, and 100 pulses, respectively. Stimulation intensity was provided at ~50% of the sensory dynamic range. This intensity was chosen to be readily detectable while minimizing the potential effects of sensory adaptation that could arise from repeated high-intensity, long-duration stimulation [44]. TDT was measured using the ascending staircase with 10ms steps, with each gap duration presented twice before moving on to the next higher gap. Two contacts were tested per session, and both contacts and three duration conditions were randomized within the session. Each condition was presented either 5 or 10 times per session, with a minimum of 10 TDT repetitions of each condition for each contact.

## Exp 3 Effect of stimulus frequency on TDT

### Exp 3.1 Frequency and TDT

We evaluated the impact of stimulation frequency on the TDT by testing four pulse train conditions delivered at 20, 40, 50, and 100 Hz. Each train lasted 500 ms and both trains in the trial were delivered at the same frequency. At the start of these sessions, the sensory dynamic range was determined for each contact using the 20 Hz condition. A stimulation level corresponding to 90% of the dynamic range was then selected and an intensity matching paradigm was used to find stimulation parameters for the other stimulation frequency conditions (i.e., 40, 50, and 100 Hz) that would yield a comparable perceived intensity to that selected for the 20 Hz condition. For intensity matching, a reference stimulus was presented, followed by a comparison stimulus, and the participant was asked if the second felt stronger, weaker, or equal in intensity to the first. The stimulation PW was adjusted in a staircase fashion until the comparison matched the reference. If a match could not be found through PW alone, the PA was incremented in 0.1 mA steps and PW was then adjusted until the reference and comparison stimuli were perceived as the same intensity.

Once stimulation parameters to yield equivalent sensation intensities across all stimulation frequencies were determined, the ascending staircase procedure with 5 ms steps was performed to find the TDT for each frequency condition. All stimuli in all trials within a staircase were presented at the same frequency. To account for the differences in inter-pulse intervals across conditions, the minimum gap tested for a given condition was equal to the pulse period for that frequency (e.g., 10 ms for 100 Hz, 20 ms for 50 Hz). In each session, two contacts were tested and the staircases were randomized across contacts and frequency conditions. Each frequency was tested 15 and 10 times per contact for P1 and P2, respectively. Normalized TDT values were computed by subtracting the minimum gap for each condition (e.g., 50 ms for 20 Hz, or 10 ms for 100 Hz) from the TDT values.

### Exp 3.2 Frequency estimation task

On the same days as those for experiment 3.1, participants completed a frequency estimation task in which they reported the perceived continuity of the pulse trains with different frequencies. Stimuli consisted of 1-s pulse trains delivered at 10, 20, 30, 40, 50, 73, and 100 Hz. For each trial, participants rated the perceived continuity of the stimulus using a Visual Analog Scale (VAS), ranging from “Fully Discontinuous” to “Fully Continuous” sensation. Here, “discontinuous” sensation meant that the participant could clearly feel the gaps within the stimulation pulse train, and “continuous” sensation meant that they did not feel any gaps within the train. Each frequency condition was repeated 20 times for each contact, and two contacts were tested in each session. Conditions and contacts were randomized in each session.

## Comparison to published TDT values for healthy subjects

To compare our measured TDT values with those previously reported for intact somatosensation, we referenced data summarized in a 2024 review by Ordas et al. [25]. Studies were included in our analysis if the review reported the mean TDT, standard deviation, and participant count for healthy subjects and if the somatosensory stimulation was applied to median innervated territory. To allow for comparison to the literature, our data was grouped into two categories: pulse TDT and train TDT. The pulse TDT group included data from Experiment 1.1 and the single pulse condition from Experiment 2.1. The train TDT group included data from Experiments 1.2 and 2.2, and the 100 Hz condition from Experiment 3.1. Only the 100 Hz condition from Experiment 3.1 was included in the train TDT group because Experiments 1.2 and 2.2 were collected at 100 Hz pulse frequency. Data was further divided into three subsets corresponding to the tested levels on the dynamic range.

## Statistical analysis

For each TDT trial, the participant’s gap judgement (i.e., gap or no gap) was recorded. These data were used to calculate the TDT value of each staircase. For TDT-based experiments (1.1, 1.2, 2.1, 2.2, 3.1), data were tested for normality using the Shapiro-Wilk test applied to each experimental condition within a participant. Homogeneity of variance was assessed using Levene’s test.

Because no datasets met the assumption of both normality and homogeneity of variance, nonparametric tests were used. Specifically, Kruskal-Wallis tests were performed to compare across stimulation conditions, followed by Dunn post hoc tests with Sidak correction for pairwise comparisons. For experiments involving repeated sessions across multiple days, datasets were assessed for variance equivalence using Levene’s test and for distribution similarity using the Kolmogorov-Smirnov test. All data was determined to be comparable and was pooled within experiment and condition between days for statistical analysis.

For Experiment 1.3, the participant’s judgement of detection (i.e., felt or not felt) was recorded. To assess detection rate differences between low and high stimulation intensities, a test of two proportions was used for each trial type (pulse or train). Additionally, each detection condition was tested against 100% (perfect detection), using Fisher’s exact test.

For Experiment 3.2, perceived continuity ratings across frequencies were first tested for normality using Shapiro-Wilk test. Because normality was not satisfied, a Kruskal-Wallis test was used to assess differences across frequency conditions, followed by Dunn-post hoc comparisons with a Sidak correction.

All analyses were performed using MATLAB R2020b (Mathworks Inc.), including functions from the Statistics and Probability Toolbox as well as a Shapiro-Wilk and Shapiro-Francia normality tests [45]. For all statistical tests, significance level was defined as α = 0.05.

## Results

### TDT decreases with increasing stimulation intensity

**Fig. 2:**
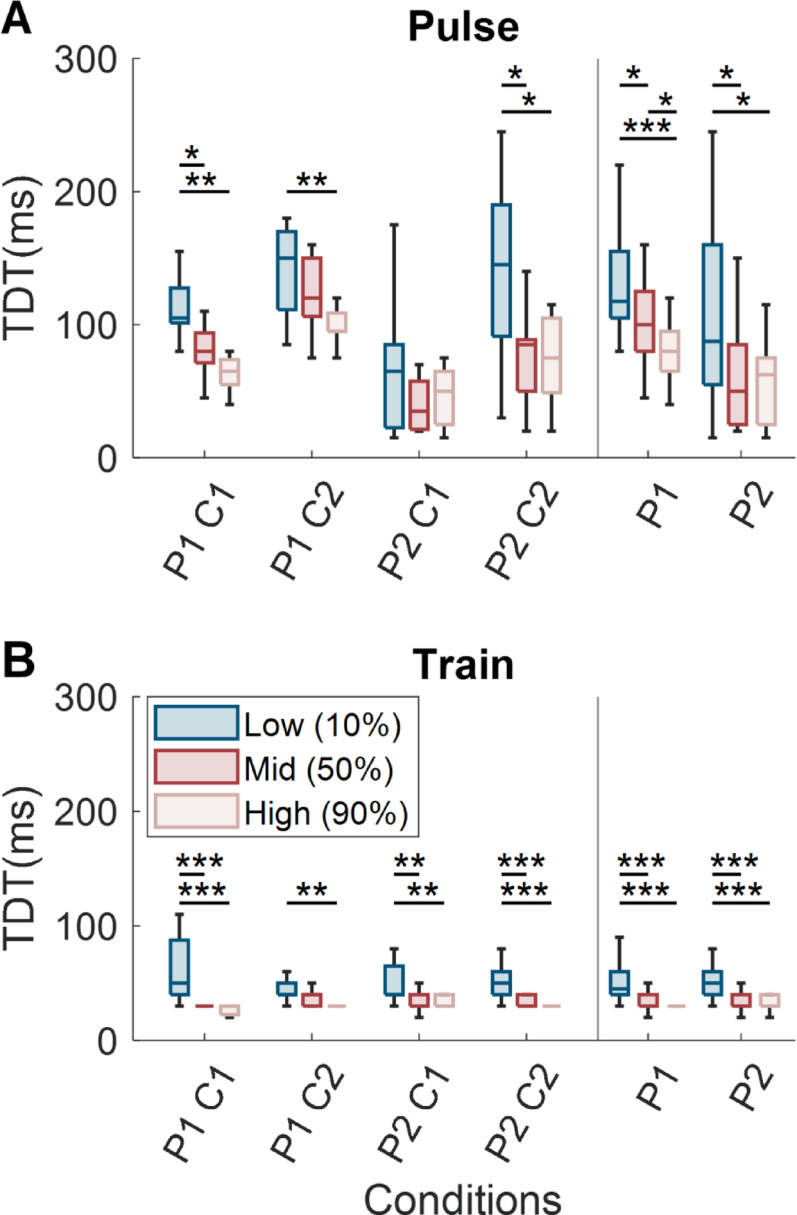
Effect of stimulation intensity on TDT for pulse trains and single pulses. **A** TDT values for 500 ms pulse trains across three intensity conditions (low, medium, high) for four contacts across two participants. **B** TDT values for pairs of single pulses across the same three intensity levels. *, **, and *** denote p < = 0.05, p < = 0.01, p < = 0.001 respectively. In each panel, the left portion depicts grouping by contact and the right depicts grouping by participant

Across both participants, TDT values were significantly higher for the low- intensity condition compared to the high- intensity condition (Fig. 2). This effect was observed for 3 of 4 contacts for pairs of single pulses (Kruskal-Wallis, *p* < 0.05; Fig. 2A) and for all contacts for pulse trains (Kruskal-Wallis, *p* < 0.01; Fig. 2B). For three of the four contacts in the pulse train experiment and two of four contacts in the single pulse experiment, the low intensity condition also resulted in significantly higher TDT values than the mid intensity condition (Kruskal-Wallis, *p* < 0.05; Fig. 2A, B). These results indicate that increasing PNS stimulation intensity reduces the TDT.

**Fig. 3:**
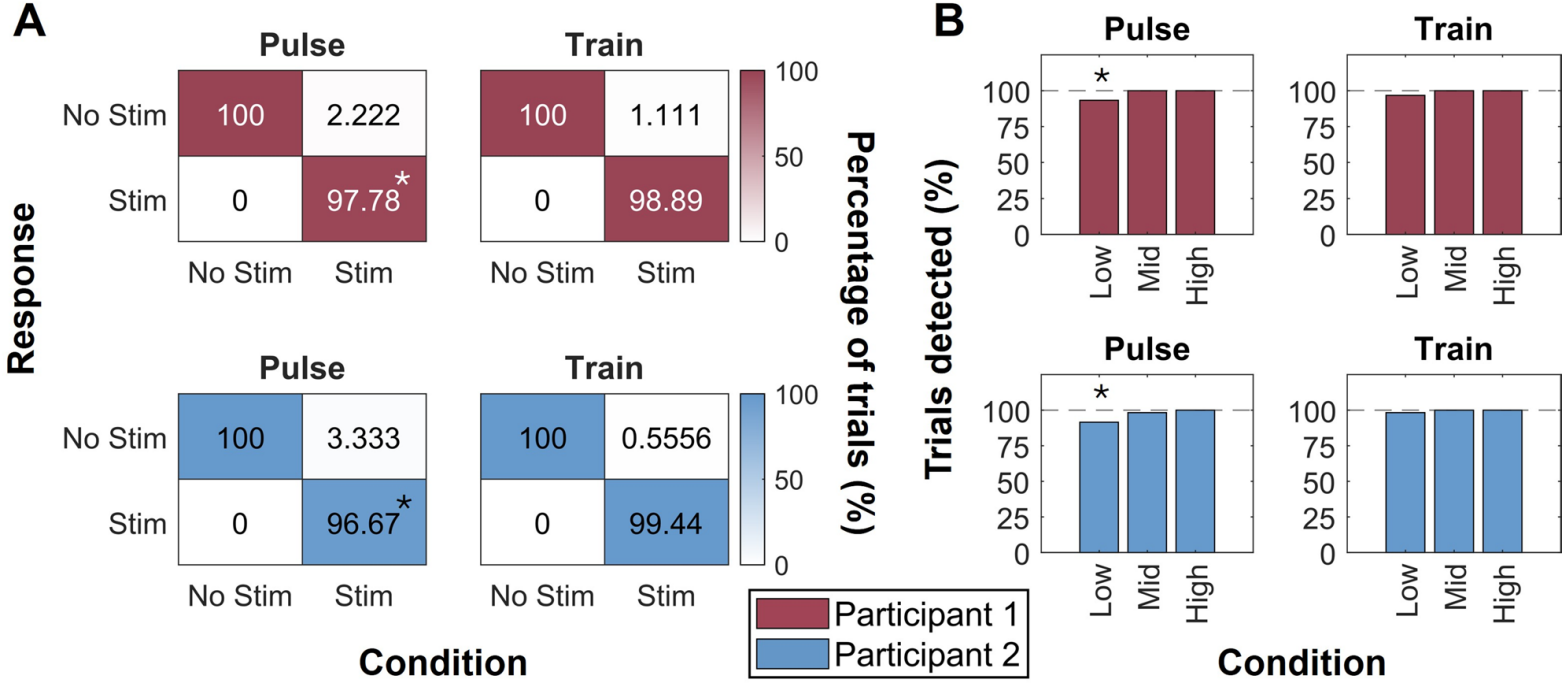
Perceptual detection of PNS pulses and trains. **A** Confusion matrices showing detection rates for single pulses (left) and pulse trains (right)). Asterisks denote conditions where performance differed significantly from 100% accuracy (*p* < 0.044). **B** Detection rates (% of trials) for each stimulation intensity condition (low, medium, and high) for single pulses (left) and pulse trains (right). The asterisk denotes a significant difference between the actual detection rate and the 100% detection rate (*p* < 0.042). Data are shown separately for P1 (top, red) and P2 (bottom, blue)

To examine whether the higher TDT values for low intensity stimuli were due to issues with detectability for these stimuli, we measured the participants’ accuracy of stimulus detection across intensity levels. In total across both participants, the stimuli were detected in 98% of trials, and no perceived sensation was reported during catch trials (Fig. 3A). Importantly, the detection rate was lower for the low intensity condition than for the medium and high intensity conditions (Fig. 3B). 

This difference was significant for both participants for single pulses (test of two-proportion *p* < 0.01) but not for either participant for pulse trains (two-proportion test, *p* > 0.05). Additionally, the detectability of the medium and high intensity conditions for both pulses and trains were not statistically different from perfect detection (Fisher’s exact test, *p* > 0.05). While the detectability of the low intensity condition was lower than the higher intensity conditions for the single pulses (Test of Two Proportions, *p* < 0.042), both participants still detected sensation on over 92.5% of low intensity pulse trials and greater than 97% of low intensity train trials.

### TDT is lower for stimulation pulse trains than for individual stimulation pulses

**Fig. 4:**
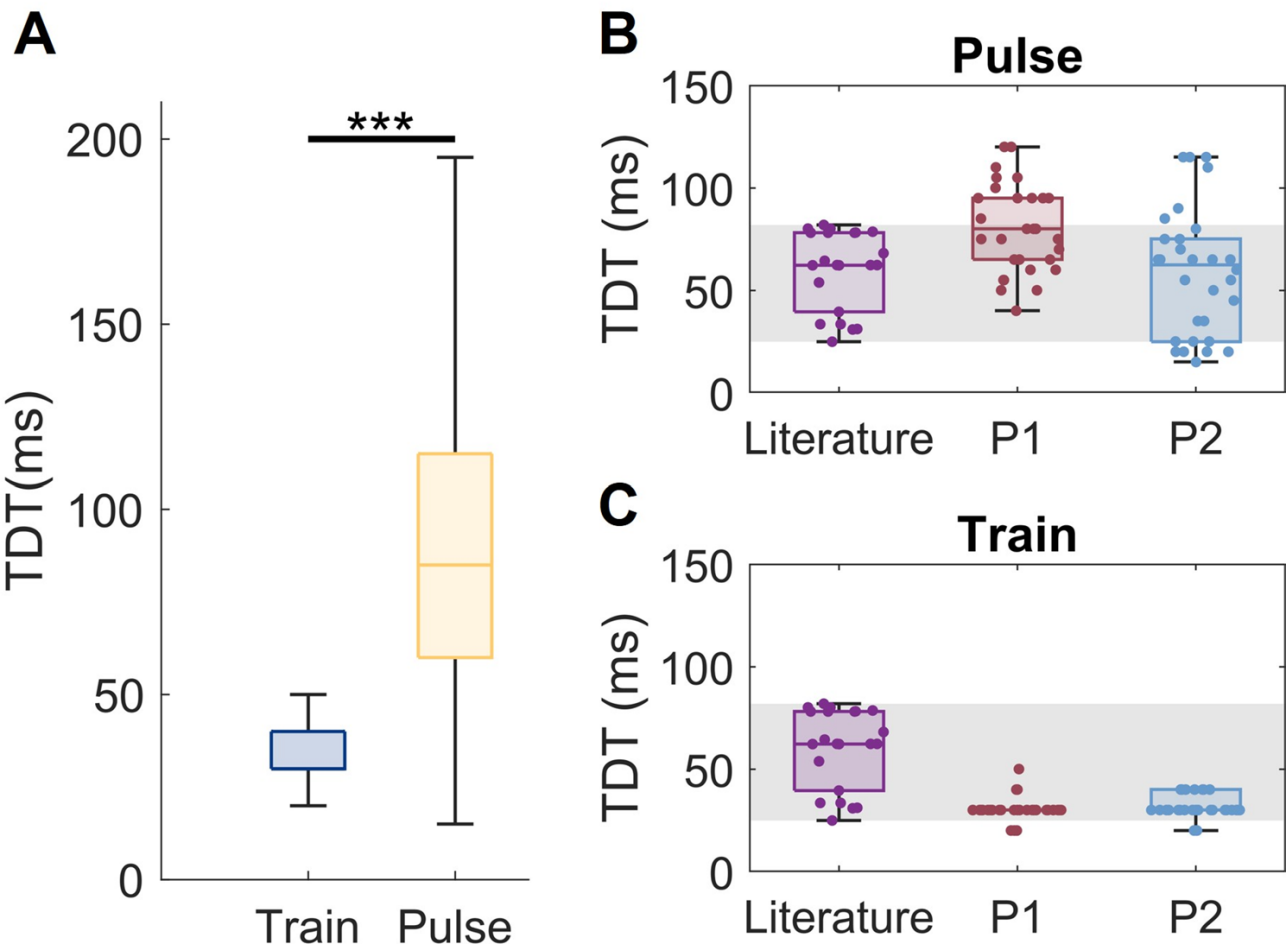
Stimulation type and TDT. **A** Difference in TDT values for pairs of pulse trains (blue) vs. pairs of single pulses (yellow). Each boxplot represents the pooled data across all intensity levels for the train or pulse condition. Asterisks denote significant difference at *p* < 0.001. B and C) TDT values for normal touch reported in a recent review (purple), where data from each individual study is shown as a circle. The shaded grey region represents the range from minimum to maximum TDT value reported in the review. **B** Pulse TDT distributions for P1 (red) and P2 (blue) for the high intensity PNS stimuli. **C** Train TDT distributions for P1 (red) and P2 (blue) for the high intensity PNS stimuli. **B** and **C** The comparisons to literature for the mid and low intensity conditions can be found in Supplemental Fig. 1

To compare pulse and train stimulation, we first performed a pooled analysis of the data from the stimulation intensity experiments and compared all pulse TDT values to all train.

TDT values. This analysis demonstrated that across sensation intensities, TDT values were significantly lower for pulse trains than for single pulses (Kruskal-Wallis, *p* < 0.001 Fig. 4A). We then compared our measured TDT values to the TDT values published in a recent review [25]. For this analysis, we compared the TDT values from the literature to our pulse condition data and train condition data separately (Fig. 4B and C, respectively). Pulse TDT values for PNS were comparable in mean and range to the TDT values reported in literature (Fig. 4B). In contrast, train TDT values for PNS were generally within the range of values reported in the literature, but were on the lower end of the range (Fig. 4C). This analysis only examined the high intensity conditions from our data, as these parameters were more similar to the intensities in the reviewed studies, given their reported methods. A comparison to the literature for our low and mid intensity TDT conditions can be found in Supplemental Fig. 1.

However, in the stimulation intensity experiments, all pulse trains consisted of 50 pulses. Given that TDT values differed between individual pulses and trains of 50 pulses (Fig. 4), we sought to further examine how pulse train duration impacted TDT. We first compared TDT values across short duration pulse trains consisting of either 1 pulse (i.e., single pulse), 2 pulses, 5 pulses, or 10 pulses. For P2, TDT values decreased with increasing pulse train duration.

**Fig. 5:**
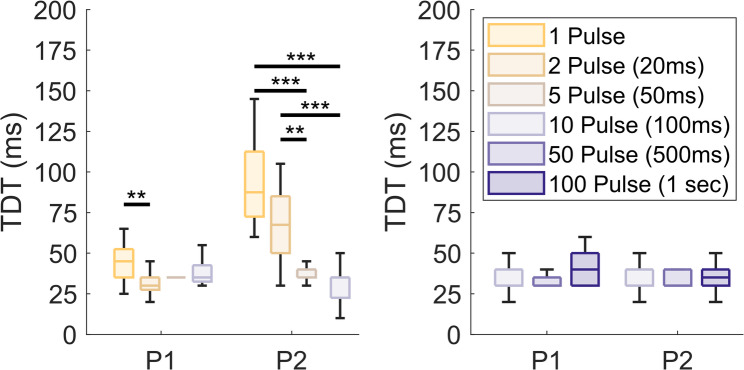
Effect of PNS stimulus duration on the TDT. **A** TDT values for short duration PNS ranging from single pulses (1 pulse, yellow) to short-duration pulse trains consisting of 2, 5, and 10 pulses per train (tan through light purple). All stimuli were intensity matched across train durations. ** and *** indicates *p* < 0.01 and *p* < 0.001, respectively. **B** TDT values for longer duration pulse trains consisting of 10, 50, and 100 pulses per train (light purple to dark purple). There were no significant differences across longer duration pulse trains

The TDT values were significantly different between 1- and 5- pulse conditions and between the 1- and 10- pulse conditions (Kruskal-Wallis, *p* < 0.001; Fig. 5A). However, P1 showed only a significant decrease in TDT between the 1- and 2- pulse conditions (Kruskal-Wallis, *p* < 0.01; Fig. 5A).

We then examined the effect of longer duration pulse trains, consisting of 10, 50, or 100 pulses. The data showed no significant difference across the 10, 50, and 100 pulse conditions for either participant (Kruskal-Wallis, *p* > 0.05; Fig. 5B).

### TDT decreases with stimulation frequency

**Fig. 6:**
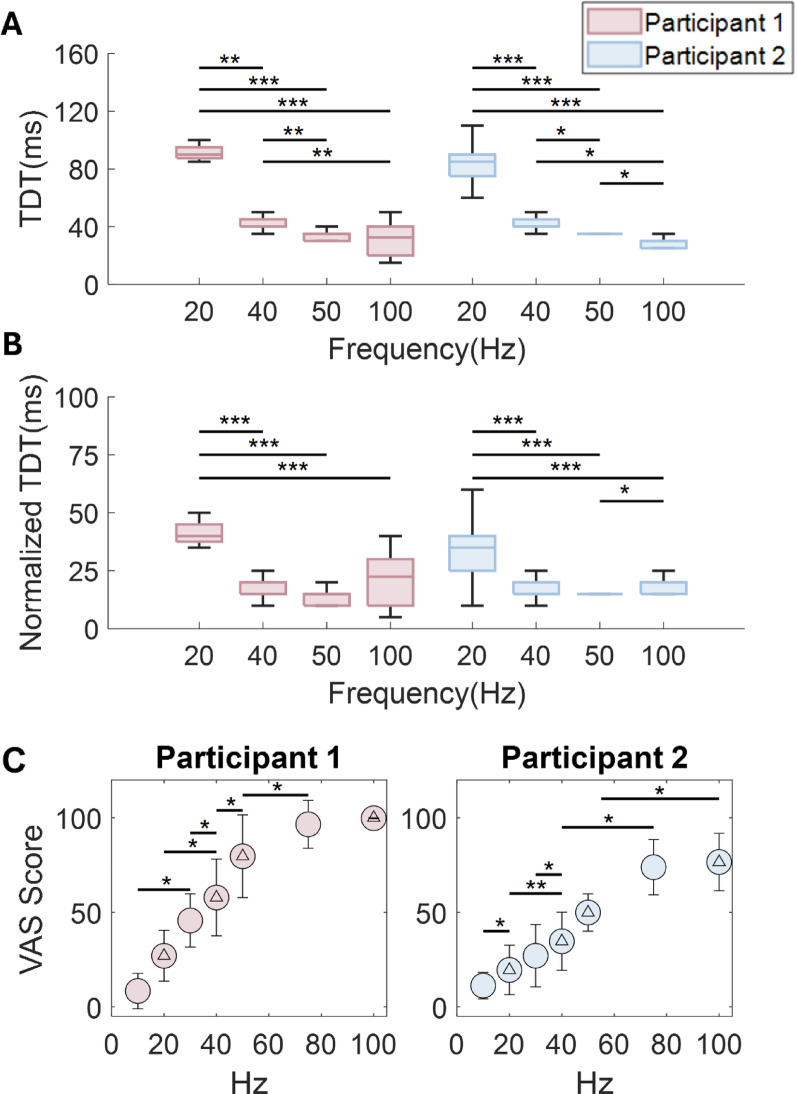
Effect of stimulation frequency on TDT. **A** Comparison of TDT values for 500ms duration, intensity-matched pulse trains that varied in pulse frequency. Significant differences were observed between the 20 Hz condition and all other conditions and between the 40 Hz condition and all other conditions for both participant 1 (red) and participant 2 (blue). There were also significant differences between the 50 and 100 Hz conditions for participant 2. **B** The TDT values were normalized across frequencies by subtracting the inter-pulse interval for each frequency, which represents the smallest possible gap for the frequency. Significant differences remained between the 20 Hz condition and all other frequencies. Significant difference also remained between 50 and 100 Hz for participant 2. All other significant differences shown in panel A were abolished. **C** The participants’ ability to perceive the frequency of PNS stimuli was assessed by asking the participants to rate the perceived continuity of the sensation (i.e., perceived temporal gaps between pulses) using a VAS slider. Continuity ratings increased with stimulation frequency, and significant differences between successive frequencies are indicated with bars and asterisks. All conditions that were two or more frequency conditions apart were significantly different (stats bars not shown to simply presentation). Frequency conditions with a triangle indicate a frequency value tested in the TDT experiments shown in panels A and B. For all panels *, **, and *** indicates *p* < 0.05, *p* < 0.01, and *p* < 0.001, respectively

We next examined how the TDT was affected by PNS pulse frequency. For both participants, TDT values decreased with increasing stimulation frequency (Fig. 6A). For both participants, TDTs at 20 Hz and 40 Hz were significantly different from all other higher frequencies (Kruskal-Wallis, *p* < 0.05). For P2, all four tested frequencies were significantly different from one another (Kruskal-Wallis, *p* < 0.042). Because we enforced that the gap between pairs of pulse trains must be greater than or equal to the gap between pulses in the train (i.e., inter-stimulus gaps must be greater than the inter-pulse interval for the train) for this experiment, we checked that the higher TDT for lower frequency pulse trains was not purely due to this requirement. We thus calculated the normalized TDT for each trial by subtracting the condition’s inter-pulse interval from the measured TDT (Fig. 6B). From this analysis, we found that the 20 Hz condition was still significantly higher than the other frequency conditions (Kruskal-Wallis, *p* < 0.001), but this normalization abolished the majority of the other significant differences among the other frequency conditions. Aside from the distinct TDT values for 20 Hz, the only other significant difference for the normalized TDT values was between the 100 Hz condition and the 50 Hz condition for participant 2 (Kruskal-Wallis, *p* < 0.05).

We also confirmed that participants could perceive differences in the stimulation frequencies via a continuity estimation task. Results from the continuity estimation task showed that ratings of perceived frequency increased monotonically with stimulation frequency (Fig. 6C). Frequency estimation ratings were also significantly different between successive frequencies for 10 Hz, 20 Hz, 30 Hz, 40 Hz, and 50 Hz in both participants (Kruskal-Wallis, *p* < 0.05), but not between the 73 and 100 Hz conditions in either participant.

## Discussion

This study demonstrated how variations in the intensity, duration, and frequency of PNS affected participants’ ability to detect temporal gaps between successive PNS stimuli that were perceived as touch on the hand. Our study builds on prior literature demonstrating the TDT for intact sensation [25, 27, 28, 41, 46], where the electrical stimulation is applied directly to the skin of the finger. Here, we report for the first time the TDT for PNS delivered via implanted interfaces in two participants with upper limb loss.

The timing of a sensory percept is a fundamental perceptual dimension, along with intensity, location, and quality. Prior studies of implanted PNS have examined how stimulation parameters, such as pulse frequency, modulated the perceived intensity [47, 48] and quality [18, 47] of evoked percepts. In the temporal domain, prior studies have examined the sensitivity of individuals to differences in temporal patterns [33] and the perception of synchrony between visual and tactile stimuli [49]. However, to our knowledge, no prior work has specifically examined sensitivity to temporal gaps in PNS-evoked sensations. Temporal gaps between sensory events may signal important information about prosthesis-object interactions during functional use, such as object slippage or changes in grip force.

In this study, we focused on three stimulation parameters - intensity, duration, and frequency - because they are commonly modulated to convey the magnitude and time course of pressure exerted by a sensorized prosthesis during grasp and manipulation tasks [50]. Since these parameters typically vary over time during prosthesis use, it is essential to understand how such variations influence a user’s ability to detect gaps between successive PNS stimuli.

### Increasing stimulation intensity improves gap detection

We found that stimulation at higher intensities correlated to lower TDT values, demonstrating that temporal delays between high-intensity stimuli are more readily detected than delays between low-intensity stimuli. This trend occurred both for pairs of individual PNS pulses and pairs of PNS pulse trains. This suggests that in sensory neuroprosthesis use cases in which the user needs to be able to detect a delay between stimuli, such as when discriminating among vibrations or textures [51], the intensity of the input will influence whether the timing discrimination is successful. To account for this relationship, future sensory encoding algorithms could either scale the stimulus’s timing with the signal’s amplitude, or a timing delay could be set to the value required for the lowest intensity stimulus, to ensure that the temporal gap is always discriminable.

The finding that stimulation intensity influenced TDT was unexpected, given that two previous studies of electro-tactile stimulation found no effect of intensity on TDT [39, 41]. One possible explanation for our finding that increasing intensity lowered TDT for implanted PNS is that participants may have been better able to detect both stimuli in the pair as stimulus intensity increased. This is because two criteria must be met for a participant to accurately perceive a temporal gap between two stimuli: (1) Both stimuli must be perceptible, and (2) The gap between the stimuli must be perceptible. If the first criterion is not met, the participant could assume that they felt no gap when, in fact, they had failed to detect one of the stimuli in the pair. The relationship between stimulus intensity and detectability is supported by classical psychophysics: psychometric functions relating stimulus intensity to the likelihood of detection demonstrate that stimuli of higher intensities are more frequently detected [48, 52]. In other words, at the detection threshold, the correct detection rate is 50%, but eventually the correct detection rate rises to 100% at sufficiently high stimulus intensities [48, 53].

We tested this “failed detection” hypothesis in experiment 1.3, where we saw that the detection accuracy of single low-intensity stimulation pulses was significantly lower than that of mid- and high-intensity pulses. However, the rates of detection were still greater than 92% for the low-intensity pulses, so the extent to which the failed detection phenomenon impacted our TDT results is unclear. We also did not run the detection and TDT experiments on the same day, so we do not know if our intensity TDT data had the same or different rates of failed detection. We selected different stimulation parameters across sessions due to small variations in the participant’s threshold and comfortable range each day, and it is possible this influenced detection accuracy. In addition, detection accuracy did not vary with intensity for the pulse trains. Thus, it is unlikely that failure of detection explains the differences between the TDT values across intensity levels for pulse trains and is only a possible explanation for the differences across intensity levels for individual pulses. Additionally, the impact of detection on the individual pulse results is less relevant for real-world neuroprosthesis applications, given that stimulation would be delivered over the entire duration that the prosthesis contacted another object, and most social interactions and object interactions occur over several seconds [54].

### The TDT of PNS pulse trains is lower than the TDT of PNS pulses

In our experiments examining the effect of stimulation intensity on TDT, we also observed that the TDTs for pulse trains were significantly smaller than TDTs for individual pulses (Fig. 4A). This implies that stimulus duration influenced TDT and that it is easier for participants to detect gaps between longer stimuli than between brief stimuli.

However, our follow-on experiments designed to investigate the effects of pulse train duration on TDT (Experiments 2.1 and 2.2) demonstrated that the relationship is not straightforward. While both participants had significantly higher TDT values for single pulses than for short pulse trains on the order of 2–10 pulses (Experiment 2.1, Fig. 5A), neither participant exhibited significant differences across pulse trains longer than 10 pulses (Experiment 2.2, Fig. 5B). Taken together, this suggests that the lower TDT for trains compared to pulses in the intensity experiments (Exp 1.1 and 1.2) would have been present even if the train durations were very brief (i.e., 2–10 pulses), since no additional TDT decreases were present when trains extended beyond 10 pulses.

It is possible that the difference between pulse and train TDT arose because participants were less confident that they perceived the PNS pulses compared to the trains, and this uncertainty led to greater difficulty in gap detection. In addition, we cannot be sure that the participants were using the same mental strategy to detect the gaps between trains as they were to detect the gaps between pulses. At longer train durations, participants may have been performing a duration estimation task rather than a gap detection task, in which they were judging the total length of the two successive stimuli against the prior set of successive stimuli. A difference in mental strategy could mean that different neural processes were being utilized for the train conditions, which may have been faster or more accurate. Ultimately, the differences between pulse and train TDT align with a growing body of literature that demonstrates that neural circuits involved in timing are different for short-duration stimuli in the range of 10 s of milliseconds versus longer-duration stimuli in the range of one to several seconds [10]. This is supported by differences in psychophysical properties such as the weber fraction [55, 56] as well as imaging studies that show duration judgment task performed on longer stimuli rely more heavily on the pre-frontal cortex in addition to the cortical regions implicated in the judgement of shorter intervals of time [57].

The methodology used for stimulation parameter selection in Experiments 1.1 and 1.2 may also have influenced the results. In Experiments 1.1 and 1.2, stimulation intensity levels were set as percentages of the sensory dynamic range measured for that day. In contrast, for Experiment 2.1 we chose a single perceptual intensity level for the single pulse condition and then adjusted the stimulation PW for the three other train durations (2, 5, and 10 pulses) to match the perceptual intensity of the single pulse condition. Because we did not map how the sensory dynamic range related to perceived intensity ratings, for Experiments 1.1 and 1.2, we do not know for sure that the low, medium, and high conditions for the single pulses were the same intensities as the respective stimulation levels for the pulse trains. Thus, we do not know what impact variations in perceived intensity between the pulse and train conditions, if any existed, may have had on the comparison between pulse and train TDT for the Exp 1.1 and 1.2 dataset. In addition, as with many other clinical neuroprosthesis studies, this study was limited by the fact that only two participants were available to perform the experiments. Thus, we do not have sufficient information to know whether the results would be more consistent with different participants or a larger participant cohort.

### Pulse train TDT is representative of neuroprosthesis use cases

 It is important to note that the clinical method to assess TDT, which is also the most pervasive approach in literature, uses individual pulses for the TDT assessment. We chose to perform the TDT measure with PNS pulse trains (in comparison to the single pulse TDT assessment approach) because this condition better emulates the duration of stimuli that would be produced by sensory neuroprostheses when interacting with an object or performing social touch [54]. Compared to prior research, the TDT values we observed for individual pulses of PNS tended to be at the upper end of previously reported TDT values or were higher than typical TDT values. In contrast, the TDT values we observed for PNS pulse trains tended to be at the lower end of previously reported values (Fig. 4). The similarity between the TDT for implanted PNS shown here and the previously reported TDT values for surface stimulation gives further support to the well-established view that the TDT is centrally mediated [25, 26, 58]. To the best of our knowledge, prior studies of TDT using vibrotactile or electrotactile stimulation typically utilize single pulses of around 200µs and have not included conditions with prolonged stimuli on the order of milliseconds to seconds, as we did in our study. Thus, much more work needs to be done to understand differences in temporal discrimination across stimulus durations for other kinds of somatosensory stimuli, such as vibrations and indentations. In addition, our findings demonstrate that neuroprosthesis developers who wish to understand the temporal discrimination capabilities of their systems should measure TDT with pulse trains rather than with the gold standard of individual pulses, as the pulse TDT results are unlikely to directly translate to real-world use cases involving longer stimuli [8, 30–32].

### Stimulation frequency influences gap detection

The frequency of stimulation is a commonly varied parameter in sensory neuroprosthesis studies, as it can be used to scale perceived intensity [48] and modulate sensation quality [18]. Additionally, there is no current standard for the stimulation frequency used in sensory encoding algorithms across research groups, with studies reporting frequency values ranging from 20 Hz to more than 200 Hz [15, 32]. Thus, we studied the impact of stimulation frequency on temporal gap detection to understand how PNS timing will vary over the frequency ranges used in neuroprosthetic applications.

In Exp. 3.1 we found that lower frequencies of stimulation had significantly higher TDT values. In addition, the differences in TDT at lower frequencies could not be explained solely by the wider intervals between pulses within the stimulation train. In other words, the increase in TDT observed at low stimulation frequencies far outpaced the increase in inter-pulse interval for these stimuli. The increase in TDT at low stimulation frequencies may be due to a difference in mental strategy to complete the TDT task at these low frequencies. Past research has shown that as the frequency of pulses in a train decreases, the sensation transitions from feeling continuous to feeling pulsatile, with the participant able to detect small temporal gaps between the individual pulses [18]. To explore how this pulsatile quality may have influenced TDT, we performed a frequency estimation task (Exp 3.2). For both participants, we found that lower frequencies were rated as more pulsatile (less continuous), with 20 Hz (the lowest frequency tested in Exp 3.1) being the most pulsatile for both participants. Thus, the participant could likely identify which TDT trials had very low frequencies due to the differences they felt in perceived frequency. When the participants felt ‘pulsatile’ or ‘non-continuous’ stimuli, they may have exhibited a bias in TDT judgements or chosen to perform a fundamentally different task during the TDT experiments. One possible strategy that could be devised specifically for pulsatile stimuli could have been comparing the durations of gaps between pulses within each pulse train with the gap between the two trains. Such a gap comparison task would likely have been much more difficult and inaccurate compared to detecting a single gap between two stimuli that were themselves perceived as continuous.

These findings show that the stimulation frequency selected for a sensory neuroprosthesis application can impact the temporal discrimination of sensory events. Our results suggest that sensory neuroprosthesis users may be better able to produce timely responses to the slippage of an object or the cessation of contact with a surface when the baseline stimulation frequency is sufficiently high (> 50 Hz), because it will allow them to more easily detect temporal gaps in sensation. In addition, implementations of neuroprostheses that use lower stimulation frequencies that are perceived to be more ‘pulsatile’ rather than perceived as a constant sensation (e.g., 20 Hz), may need to balance the benefits of this setting, such as reduced motor activation [59], with a participant’s reduced ability to detect gaps or breaks in stimulation trains at these frequencies. For sensory neuroprostheses that use stimulation frequency to scale percept intensity and/or quality, smart algorithms could be designed that automatically update the stimulation frequency to achieve the desired intensity and/or quality while also allowing for temporal features to be discernible.

### Temporal discrimination in object slippage and biomimetic PNS paradigms

The ability to detect and quickly react to object slippage is key in day-to-day interactions with objects, and thus effective neuroprostheses will need to convey object slippage resulting from a weak or unsteady interaction between a prosthesis and an object. Incipient object slip can be detected by shear sensors [60] or piezoelectric sensors that detect high frequency signals indicative of slippage events [61–63]. A prosthesis equipped with these sensors could trigger a specific PNS paradigm which warns the prosthesis user about the object slip. In laboratory settings, slip information has been conveyed using temporally varying pulse trains delivered to two contacts [64], as well as with high intensity discrete pulses from a single contact [60]. Understanding the TDT of PNS allows for an approach where the ongoing real-time stimulation conveying the temporal profile of pressure from contact with an object is interrupted for a brief delay that is long enough to convey a distinct gap, which serves as a slip indicator. An algorithm for this slip alert paradigm would take into account the frequency and intensity of the ongoing stimulation to calculate the needed duration of a gap to be readily detectable by the user while still being brief enough for the user to respond rapidly to the slip event. This gap-based approach allows for the slip alert to be perceptually distinct from sensation intensity, which is typically used as an indicator of pressure of contact between the prosthesis and object in home-use studies of sensorized neuroprosthesis [32, 65].

Understanding how intensity and frequency shape TDT could also provide insight into the development of biomimetic stimulation algorithms. Biomimetic approaches vary the intensity and/or frequency [66, 67] of pulses during a stimulation event to better approximate natural afferent fiber activation during normal touch. Studies show that biomimetic stimulation can improve the naturalness [67] of sensation as well as improve reaction times during prosthesis control [66, 67]. Given that the present study demonstrated that variations in frequency and intensity can influence the temporal discrimination of PNS stimuli, more work is needed to understand the TDT for biomimetic PNS paradigms, where temporal variations in stimulation parameters can occur rapidly within a single touch event. Nonetheless, results from this study suggest that the temporal delays between the various phases in the biomimetic stimulation paradigm must be less than the TDT for biomimetic stimuli to feel like one continuous touch rather than multiple separate touch events.

## Conclusion

This study improves our understanding of the resolution of temporal gap detection for tactile sensations produced by direct stimulation of the nerves via implanted interfaces, expanding our understanding of the perceptual properties of PNS [33, 51, 68]. The ability to detect gaps between PNS-elicited tactile stimuli has important implications for the ability to perform neuroprosthetic tasks that involve detection and response to rapid changes in tactile inputs, such as texture perception, grating identification, stereognosis, or object slip prevention. This study also provides insight into how variations in the PNS-elicited neural code - due to changes in stimulation intensity, duration, and frequency - impact these temporal properties [21, 25, 33]. Because stimulation intensity, duration, and frequency will likely covary during neuroprosthesis use to convey other perceptual properties, future work should explore how these interactions affect neuroprosthesis experience and functional outcomes. Further development of our understanding of the temporal properties of PNS-evoked sensation is key to the effective translation of sensory neuroprosthesis technology setting the stage for widespread adoption of these devices amongst prosthesis users.

## Supplementary Information

Supplementary Material 1.

## Data Availability

The datasets used and/or analyzed during the current study are available from the corresponding author on reasonable request.
