# Supplementary material for: Mind the gap: the temporal discrimination threshold of tactile sensation from implanted peripheral nerve stimulation
Source: J Neuroeng Rehabil. 2026 Apr 18;23:179. doi: 10.1186/s12984-026-01986-9 (PMC13235127; doi:10.1186/s12984-026-01986-9)
Supplement: Supplementary file 1 — Supplementary Material 1. [file 12984_2026_1986_MOESM1_ESM.docx]

Appendix 1

Comparison to Existing Literature

TDT values from our experiments were compared with existing literature values taken from the review by Oddo et al. Figure X in the main text outlines the relationship between our “high” intensity cases and the reported literature. This was selected as the thresholding and stimulation selections reported in standard TDT lecture likely approximated the higher stimulation settings. Below is the analysis repeated on all the data as well as the low and mid intensity settings.

Appendix-Figure 1- Comparison to Literature Values- The full comparison of Pulse (A-C) and Train (E-F) TDT values between the Intensity tests and literature values. For all panels literature values(purple) were taken from the review by Oddo et al based on the described methods. Values for all 3 intensities (Low, Mid and High) are displayed in (A,D) for the pulse and train respectively. The medium intensity values are shown in panels B,D. The low intensity in C,F. The high intensity cases are shown I the main manuscript.

A

B

C

D

E

F

Appendix 2: Threshold and SDR values from Exp 1.1-1.3.

Table 2. Summary of thresholds and stimulation levels across participants, contacts, and sessions. For each contact (rows), mean and standard deviations are given for the following parameters: PW at detection threshold, PW at 10% of the sensory dynamic range (SDR), PW at 50% of the SDR, and PW at 90% of the SDR. The 10%, 50%, and 90% PW levels correspond to the Low, Mid and High intensity conditions in for each repetition of all sub-experiment of Experiment 1.

| Participant | Contact | Threshold (µs) | 10% SDR (µs) | 50% SDR (µs) | 90% SDR (µs) |
| --- | --- | --- | --- | --- | --- |
| Participant 1 | C1 | 60±18 | 70±20 | 114±20 | 115±27 |
| Participant 1 | C2 | 32±7 | 38±5 | 72±8 | 106±15 |
| Participant 2 | C1 | 30±5 | 44±11 | 100±36 | 156±63 |
| Participant 2 | C2 | 45±15 | 62±19 | 125±37 | 185±57 |
